# Differences in Involvement of Whole-Body Compensatory Alignment for Decompensated Spinopelvic Sagittal Balance

**DOI:** 10.3390/jcm12144690

**Published:** 2023-07-14

**Authors:** Jun Ouchida, Hiroaki Nakashima, Tokumi Kanemura, Kenyu Ito, Mikito Tsushima, Masaaki Machino, Sadayuki Ito, Naoki Segi, Yukihito Ode, Shiro Imagama

**Affiliations:** 1 Department of Orthopaedics, Nagoya University Graduate School of Medicine, Nagoya 464-8601, Japan; 2 Department of Orthopedic Surgery, Konan Kosei Hospital, Konan 483-8704, Japan

**Keywords:** sagittal alignment, knee flexion, compensation parameter, spinopelvic sagittal balance, age-related change

## Abstract

Background: The aim of this study was to investigate the differences in the involvement of whole-body compensatory alignment in different conditions of spinopelvic sagittal balance (compensated/decompensated). Methods: We enrolled 330 individuals who underwent medical checkups and divided them according to sagittal vertical axis (SVA): for the compensated group, this was <4 cm, (group C) and for the decompensated group, it was ≥4 cm, (group D). The correlation between the lack of ideal lumbar lordosis (iLL), which was calculated by using the Schwab formula, and the compensatory radiographic parameters in each group was analyzed. The threshold value of knee flexion (KF) angle, which indicated spinopelvic sagittal imbalance (SVA ≥ 4), was determined by a ROC-curve analysis. Results: The correlation analysis of the lack of iLL and each compensatory parameter showed a strong correlation for pelvic tilt (PT) (r = −0.723), and a weak correlation for thoracic kyphosis (TK) (r = 275) in Group C. In Group D, the correlations were strong for PT (r = −0.796), and moderate for TK (r = 0.462) and KF (r = −0.415). The optimal cutoff value for the KF angle was determined to be 8.4 degrees (sensitivity 89%, specificity 46%). Conclusions: The present study shows differences between compensated/decompensated spinopelvic sagittal balance in the correlation strength between lack of iLL and whole-body compensatory parameters.

## 1. Introduction

Whole-body balance in the standing posture with a horizontal gaze is based on the alignment chain from the head to the feet, and it is well known that degenerative changes in spinopelvic alignment cause compensatory changes in alignment, including in the lower extremities [1,2,3]. Several studies have shown that degenerative changes in the spine begin with a decrease in lumbar lordosis (LL) and progress, eventually, to spinopelvic imbalance, which leads to a reduction in quality of life [2,3,4,5]. The sagittal vertical axis (SVA) is a spinopelvic sagittal-plane parameter that is used to measure the offset between a plumb line from C7 to the pelvis, and it has been widely used as an indicator of spinopelvic sagittal balance in the treatment of patients with spinal deformities [1,4,6].

Knee flexion (KF) is an effective compensatory mechanism to handle changes in spinopelvic sagittal alignment [3,5], and patients with severe spinopelvic imbalance attempt to maintain standing balance by flexing their knee joints [4,7]. This requires excessive activity of the quadriceps and psoas muscles to maintain a standing posture and is not an economical state [4]. In addition, the importance of spinopelvic sagittal imbalance on whole-spine radiographs is minimized by KF in patients with severe degenerative-spine conditions [3]. The evaluation of KF is considered necessary when assessing spinopelvic sagittal balance in the treatment of patients with spinal deformities.

There is no doubt that KF and pelvic rotation are effective compensatory mechanisms for handling changes in spinopelvic alignment in the standing position [8,9,10]. However, few studies have addressed whole-body compensatory mechanisms in response to degenerative changes in spinopelvic sagittal balance in large healthy cohorts with no history of spinal treatment. The differences in the behavior of compensatory alignment, including in the lower extremities, between compensated spinopelvic sagittal balance and the decompensated state are still unclear. We hypothesized that if the specific recruitment of KF to compensate for degenerative changes in spinopelvic sagittal balance were to be clarified, the threshold value of KF angle indicating decompensated spinopelvic balance would be a useful indicator for the treatment of spinal deformity. The purposes of this study were, first, to show the differences in the involvement of whole-body compensatory alignment changes, including in the lower extremities, in each condition of compensated and decompensated spinopelvic sagittal balance, and, second, to determine the cutoff value for the KF angle that suggests spinopelvic sagittal imbalance.

## 2. Materials and Methods

### 2.1. Subjects

We enrolled 330 subjects from the spine-medical-checkup database at our hospital. Subjects with any past and/or current medical history of spinal disease, neurological disease, or treatment for hip- or knee-joint disease affecting assessment in the standing posture were excluded from this cross-sectional, observational analysis.

Responses to a questionnaire and the Oswestry disability index (ODI) score were obtained from all participants to evaluate their clinical complaints, and they all underwent whole-body X-ray using a scanning-X-ray-imaging system (EOS Imaging, Paris, France). The examination posture was a “hands-on-cheek” posture with the participants’ fingers lightly touching in the standing position with a horizontal gaze. The participants were instructed to relax as much as possible during the X-ray.

This study was approved by the local Institutional Review Board, and written informed consent was waived because of the retrospective design.

### 2.2. Whole-Body Sagittal-Plane Parameters

The SterEOS software program (SterEOS 1.6, Postural Assessment Workflow, EOS Imaging) was used to measure the radiographical parameters. Sagittal-plane alignment in the whole body from cranio-cervical junction to ankle joint was analyzed in this study. The following parameters were analyzed: occipito-C2 angle (O-C2 angle: McGregor line–C2 endplate), C2–7 lordotic angle (C2 endplate–C7 caudal endplate), T1 slope, thoracic kyphosis (TK: T1–12), LL (L1-S1), sacral slope (SS), pelvic tilt (PT), pelvic incidence (PI), KF angle (average of left and right KF angles; KF angle was measured as the angle between the femoral axis and the tibial axis, the line connecting the intercondylar fossa of the femur and the center of the inferior articular surface of the tibia), and ankle angle (AA: tibia shaft-vertical line) as spinopelvic- and lower-extremity-alignment parameters; and SVA, CAM-HA/knee/ankle offset (the distance in a plumb line from the acoustic meatus to the center of the femoral head/knee joint/ankle joint), and T1 pelvic angle (TPA), as global balance parameters. Kyphosis and lordosis were defined as the angle between the upper endplate of a selected vertebra and the lower endplate of another selected vertebra. The correlation between KF and SVA in all the subjects enrolled in this analysis was investigated.

### 2.3. Correlations between Lack of Ideal Lumbar Lordosis and Whole-Body Compensation Alignment

We divided the subjects into two groups, according to the compensation status of spinopelvic sagittal balance. The compensation status was determined by SVA; subjects with a measured SVA of <4 cm were assigned to the compensated group (group C), and those with a SVA ≥ 4 cm were assigned to the decompensated group (group D). The cutoff for SVA was the threshold for anterior spinal inclination in the spinal-deformity classification proposed by Schwab et al. [11] Patient demographic data, including age, sex, height, weight, and ODI [12], and the radiographic parameters described above were compared between these two groups.

We calculated the ideal lumbar lordosis (iLL) from the measured spinopelvic parameters with reference to the formula proposed by Schwab et al. [6,13] and analyzed the correlation of lack of iLL with the compensatory parameters to clarify the recruitment of the compensatory mechanisms in each group: lack of iLL = measured LL − (PI + 9). The compensatory parameters included O-C2 angle, C2–7 lordotic angle, T1-slope, TK (T1–12), SS, PT, KF, and AA.

We determined the threshold of KF angle that suggested recruitment of knee-joint flexion indicative of a spinopelvic sagittal imbalance by drawing receiver operatoing characteristic (ROC) curves and performing a cutoff analysis. The existence of spinopelvic sagittal imbalance was defined as SVA > 4 cm, and an ROC curve for KF was drawn. The area under the curve (AUC) was calculated, and a cutoff value for KF angle indicating spinopelvic sagittal imbalance was determined at the coordinate point with the maximum sum of sensitivity and specificity.

### 2.4. Statistical Analysis

All values are expressed as the mean ± standard deviation. The Mann–Whitney U test was used to compare demographic data and radiographic parameters of the two compensation groups. Pearson’s correlation coefficient (r) was used to show the correlation between KF and SVA and to measure the strength of correlations between lack of iLL mismatch and each compensatory parameter. The strength of the correlation between each parameter was described using the absolute value of r (r = 0.20–0.39: weak, r = 0.40–0.59: moderate, r = 0.60–0.79: strong, and r = 0.80–1.0: very strong correlation). The *p* values of <0.05 were considered statistically significant. The IBM SPSS Statistics version 23.0 (IBM Corp., Armonk, NY, USA) was used for the statistical analyses.

## 3. Results

**Figure 1:**
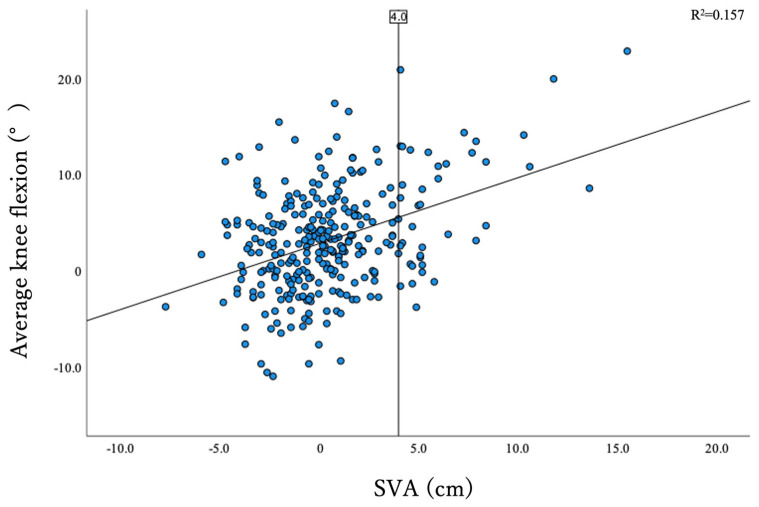
Dot plot showing the correlation between SVA and KF angle. Vertical line indicates SVA = 4. KF, knee flexion; SVA, sagittal vertical axis.

After the exclusion of the subjects meeting the exclusion criteria, 294 subjects were included in the statistical analysis. Their average age was 58.0 ± 12.7 years; 59.9% of the subjects were male (n = 176), and their average ODI score was 11.0 ± 10.2. The overall correlation between SVA and KF was r = 0.396 (*p* < 0.001). A dot plot displaying the correlation between SVA and KF is shown in Figure 1. There were 253 patients in group C with compensated spinopelvic sagittal balance (SVA < 4) and 41 patients in group D with spinopelvic sagittal imbalance (SVA ≥ 4). The demographic data and radiographic parameters of the two groups are compared in Table 1. There were significant differences between group C and group D, respectively, in age (56.7 ± 12.8 and 65.7 ± 9.5 years, *p* < 0.001), height (162.2 ± 9.1 and 159.3 ± 9.0 cm, *p* = 0.043), ODI score (10.1 ± 9.7 and 16.8 ± 11.4, *p* = 0.001), C2–7 lordotic angle (1.3 ± 11.3 and 8.2 ± 10.2 degrees, *p* < 0.001), T1-slope (23.3 ± 8.6 and 29.2 ± 8.2 degrees, *p* < 0.001), LL (47.4 ± 12.0 and 35.3 ± 18.1 degrees, *p* < 0.001), lack of iLL (−1.4 ± 10.2 and −17.3 ± 15.9 degrees, *p* < 0.001), PT (14.2 ± 7.1 and 20.9 ± 10.2 degrees, *p* < 0.001), KF (2.5 ± 5.0 and 7.3 ± 6.5 degrees, *p* < 0.001), AA (5.3 ± 3.0 and 6.6 ± 3.3 degrees, *p* = 0.028), SVA (−0.4 ± 2.1 and 6.2 ± 2.7 cm, *p* < 0.001), CAM-HA (−2.4 ± 2.9 and 2.5 ± 3.9 cm, *p* < 0.001), CAM-knee offset (−0.2 ± 2.9 and 2.9 ± 3.8 cm, *p* < 0.001), CAM-ankle offset (2.7 ± 3.0 and 6.8 cm ± 3.4, *p* < 0.001), and TPA (9.3 ± 6.5 and 21.0 ± 11.0 degrees, *p* < 0.001).

The correlation analysis of the lack of iLL with each compensatory parameter showed a strong correlation for PT (r = −0.723, *p* < 0.001), a weak correlation for TK (r = 275, *p* < 0.001) and SS (r = 0.267, *p* < 0.001), and very weak correlations for KF (r = −0.153, *p* = 0.015) and AA (r = −0.129, *p* = 0.040) in Group C. In Group D, the correlations were strong for PT (r = −0.796, *p* < 0.001), moderate for TK (r = 0.462, *p* = 0.002), SS (r = 0.577, *p* < 0.001) and KF (r = −0.415, *p* = 0.007), and weak for AA (r = −0.334, *p* = 0.033) (Table 2).

**Figure 2:**
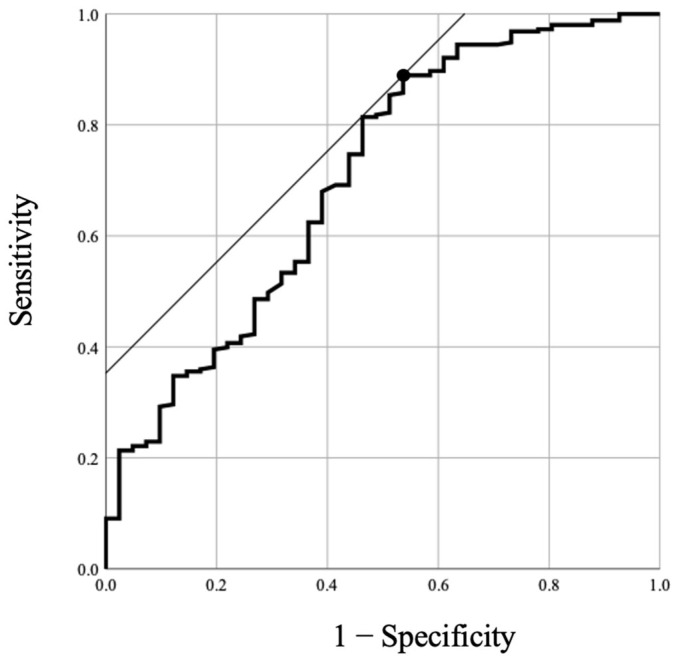
ROC curve of KF angle for prediction of spinopelvic sagittal imbalance (SVA > 4). AUC was 0.704 (95% confidence interval, 0.61–0.80). The cutoff point for KF was 8.4° (sensitivity, 89%; specificity, 46%). ROC, receiver operating characteristic; SVA sagittal vertical axis; AUC area under the curve; KF knee flexion.

The ROC curve of the KF angle for the prediction of spinopelvic sagittal imbalance (SVA > 4) is shown in Figure 2. The AUC was 0.704 (95% confidence interval, 0.61–0.80). The optimal cutoff value for the KF angle was determined to be 8.4 degrees, which maximized the sum of the sensitivity (89%) and specificity (46%).

## 4. Discussion

In this study, the differences in correlation strength between the lack of lumbar lordosis and the whole-body compensatory parameters of the different compensatory phases of spinopelvic sagittal balance were shown in a relatively large cohort of subjects with no history of spinal treatment. A comparative analysis of the compensatory and decompensatory groups revealed that during the decompensatory phase of spinopelvic sagittal balance, the standing posture was maintained by the more intense recruitment of whole-body compensatory parameters from the spine to the feet, excluding craniocervical and cervical alignment. There was a significant correlation between the SVA and KF angles in the overall subject population. In contrast, the correlation between the lack of iLL and the KF angle was very weak in group C, with compensated spinopelvic sagittal balance.

As LL decreases with spinal degeneration, the discrepancy between LL and PI, which is considered a constant, becomes larger [2,14]. As long as spinal deformity can be compensated, spinopelvic sagittal balance is maintained by the compensatory mechanism in the spine [3,8]. The initial major compensatory mechanism at work in the early phase of this change in spinal alignment is an increase in PT due to posterior pelvic retroversion [9,15]. Similarly, in the present study, we found a strong correlation between a lack of iLL and PT in Group C, with compensated spinopelvic sagittal balance.

With the progression of degeneration, the lack of iLL becomes greater, and as spinopelvic sagittal balance declines into the decompensated phase, compensatory alignment changes in the whole body develop [15]. In group D, with decompensated spinopelvic sagittal balance, we found a moderate correlation between lack of iLL and the recruitment of KF, and a weak correlation between Llack of iLL and ankle dorsiflexion in the maintenance of a standing posture. The KF is considered an effective compensatory mechanism, and previous studies showed that ankle flexion is also an important compensatory mechanism, especially in the elderly [5,7,16]. Obeid et al. [5] reported a correlation between lack of theoretical LL and KF in a study of patients with major spinal deformities. Diebo et al. [8] analyzed 161 adult patients with sagittal spinal malalignment and reported that as the PI-LL increased, the contributions of TK and PT to the compensation cascade decreased and those of the KF angle and pelvic shift increased. The correlation between lack of iLL and the KF angle was moderate in Group D, which supports the findings of these other studies. However, the analyses in these previous studies were limited to patients with spinal deformities, whereas the present study is the first, to our knowledge, to analyze a series of patients with no history of spinal treatment and to calculate the threshold for the KF angle that indicates spinopelvic sagittal imbalance.

In the cutoff analysis using the ROC curve, we found that the KF angle indicating spinopelvic imbalance was 8.4 degrees. Hasegawa et al. [15] classified their cohorts, which included healthy subjects and patients with spinal deformities, according to health-related quality of life (HRQOL) and reported significant differences in TPA, C2–7 kyphosis, PI-LL, PT, and KF between these groups. They also reported that the threshold value of the KF angle for severe disability was 8 degrees, which is very similar to the cutoff value for the KF angle determined in the present study. These findings, including the KF cutoff and the observed relationship between the KF and global spinopelvic sagittal alignment, have potential for use in the screening of patients undergoing knee-plane radiographs to identify spinopelvic sagittal-imbalance conditions. Moreover, we believe that these findings will serve as a more convenient index for evaluating global balance and assessing treatment outcomes in patients with spinal deformities, particularly in cases in which there may be challenges in recognizing radiographic parameters in the sacral pelvic region [17,18]. However, the compensatory mechanisms of the whole body consist of many alignments in mobility [3,5], and it is uncertain whether a single variable, such as KF, can accurately reflect an individual patient’s state of balance maintenance. Additional validation studies are needed to determine whether the cutoff value for the KF in the present study is a relevant radiographic indicator of preferable outcomes related to HRQOL in the treatment of adult subjects with spinal deformities.

A limitation of this study is the lack of information on degenerative knee-joint disease and the range of motion of the knee because of the retrospective study design. The subjects in Group D with spinopelvic sagittal imbalance were older, and we cannot rule out the possibility that osteoarthritis (OA) or other diseases affecting knee-joint range of motion might have affected the results of the analysis [19]. Furthermore, the small number of participants classified as group D and the lack of anatomical power are further limitations of this study. Future multivariate analyses, including a larger number of cases, may be necessary to discern the association between global sagittal imbalance and knee flexion, as well as to evaluate the influence of age-related factors (e.g., gluteal -muscle strength and joint OA) on this association [20,21].

## 5. Conclusions

In the present study, we investigated the differences in the correlation strength between lack of iLL and whole-body compensatory parameters between the two different phases of spinopelvic sagittal balance. In the subjects with compensated spinopelvic balance, there was a strong correlation between lack of iLL and PT and a weak correlation between lack of iLL and TK. In the subjects with decompensated spinopelvic sagittal balance, there was a stronger correlation between lack of iLL and TK, and between the recruitment of KF and that of AA. These findings showed differences between compensated and decompensated spinopelvic sagittal balance in the correlation strength between lack of iLL and whole-body compensatory parameters. The cutoff value for the KF angle indicative of decompensated spinopelvic balance from the ROC analysis was 8.4 degrees. Future studies are needed to establish whether the cutoff value determined in this study can be used as a potential outcome measure in the treatment of spinal deformities.

## Figures and Tables

**Table 1:** The overall demographic and radiographical parameters and those of each group.

	Overall	Compensated (Group C)	Decompensated (Group D)	*p*
Cases, n	294	253	41	
Age, years	58.0 ± 12.7	56.7 ± 12.8	65.7 ± 9.5	<0.001
Sex, Male, %	176 (59.9%)	151 (59.9%)	25 (61.0%)	
Height, cm	161.8 ± 9.1	162.2 ± 9.1	159.3 ± 9.0	0.043
Weight, kg	59.8 ± 12.1	60.1 ± 12.3	57.8 ± 10.0	0.254
BMI, kg/m^2^	22.5 ± 4.0	22.5 ± 4.0	22.7 ± 3.4	0.858
ODIscore, %	11.0 ± 10.2	10.1 ± 9.7	16.8 ± 11.4	0.001
Radiographical parameters				
O-C2 angle, degrees	16.4 ± 7.9	16.3 ± 8.0	16.8 ± 7.0	0.990
C2–7 lordotic angle, degrees	2.3 ± 11.4	1.3 ± 11.3	8.2 ± 10.2	<0.001
T1-slope, degrees	24.1 ± 8.7	23.3 ± 8.6	29.2 ± 8.2	<0.001
TK (T1–12), degrees	40.5 ± 12.0	41.1 ± 11.9	36.8 ± 11.7	0.061
LL (L1-S1), degrees	45.7 ± 13.6	47.4 ± 12.0	35.3 ± 18.1	<0.001
lack of iLL, degrees	−12.6 ± 12.5	−10.4 ± 10.2	−26.3 ± 15.9	<0.001
SS, degrees	33.7 ± 9.5	34.2 ± 8.8	30.7 ± 12.7	0.066
PT, degrees	15.1 ± 8.0	14.2 ± 7.1	20.9 ± 10.2	<0.001
PI, degrees	49.3 ± 10.9	48.8 ± 10.5	52.6 ± 12.8	0.109
KneeFlex, degrees	3.2 ± 5.5	2.5 ± 5.0	7.3 ± 6.5	<0.001
AA, degrees	5.5 ± 3.1	5.3 ± 3.0	6.6 ± 3.3	0.028
SVA, cm	0.5 ± 3.2	−0.4 ± 2.1	6.2 ± 2.7	<0.001
CAM-HA, cm	−1.7 ± 3.5	−2.4 ± 2.9	2.5 ± 3.9	<0.001
CAM-knee offset, cm	0.2 ± 3.2	−0.2 ± 2.9	2.9 ± 3.8	<0.001
CAM-ankle offset, cm	3.3 ± 3.3	2.7 ± 3.0	6.8 ± 3.4	<0.001
TPA, degrees	11.0 ± 8.3	9.3 ± 6.5	21.0 ± 11.0	<0.001

BMI, body-mass index; ODI, Oswestry disability index; TK, thoracic kyphosis; LL, lumbar lordosis; PI, pelvic incidence; SS, sacral slope; PT, pelvic tilt; AA, ankle angle; SVA, sagittal vertical axis; CAM-HA, center of acoustic meatus and center of femoral-head offset; CAM-K, CAM, and center of the knee; CAM-A, CAM, and center of the ankle joint; TPA, T1 pelvic angle.

**Table 2:** Results of correlational analysis of compensations for lack of iLL.

**Compensated (Group C)**								
	**O-C2 Angle**	**C2–7 Lordotic Angle**	**T1-Slope**	**TK(T1–12)**	**SS**	**PT**	**KF**	**AA**
Pearson’s Correlation Coefficient	0.002	−0.017	0.016	0.275 **	0.267 **	−0.723 **	−0.153 *	−0.129 *
*p*	0.978	0.782	0.795	<0.001	<0.001	<0.001	0.015	0.040
**Decompensated (Group D)**								
	**O-C2 Angle**	**C2–7 Lordotic Angle**	**T1-Slope**	**TK(T1–12)**	**SS**	**PT**	**KF**	**AA**
Pearson’s Correlation Coefficient	0.088	−0.297	−0.180	0.462 **	0.577 **	−0.796 **	−0.415 **	−0.334 *
*p*	0.585	0.059	0.261	0.002	<0.001	<0.001	0.007	0.033

The strength of the correlations between each parameter were as follows: r = 0.20–0.39, weak; r = 0.40–0.59, moderate; r = 0.60–0.79, strong; and r = 0.80–1.0, very strong correlation. PI, pelvic incidence; LL, lumbar lordosis; TK, thoracic kyphosis; SS, sacral slope; PT, pelvic tilt; KF, knee flexion; AA, ankle angle. * denotes correlations with significance. ** denotes correlations equal to or stronger than weak correlation.

## Data Availability

The datasets generated and/or analyzed during the current study are available from the corresponding author on reasonable request.
